# Person‐directed care planning in nursing homes: A scoping review

**DOI:** 10.1111/opn.12212

**Published:** 2018-10-25

**Authors:** Michael Lepore, Kezia Scales, Ruth A. Anderson, Kristie Porter, Trini Thach, Eleanor McConnell, Kirsten Corazzini

**Affiliations:** ^1^ RTI International Washington District of Columbia; ^2^ Duke University School of Nursing Durham North Carolina; ^3^ The Office of Research Support and Consultation (RSC) University of North Carolina‐Chapel Hill School of Nursing Chapel Hill North Carolina; ^4^ RTI International Research Triangle Park North Carolina; ^5^ Present address: PHI Bronx New York; ^6^ Present address: Department of Health Policy and Management University of North Carolina Chapel Hill North Carolina

**Keywords:** care planning, nursing home care, person‐centred care, person‐directed care

## Abstract

**Aim:**

Federal regulations require nursing homes in the United States to support residents in directing their own care rather than having their care plans developed for them without their engagement, but knowledge of person‐directed approaches to care planning in nursing homes is limited. The purpose of this study was to advance understanding of person‐directed care planning (PDCP).

**Methods:**

A multidisciplinary research team conducted a scoping review on individual and family involvement in care planning, including literature from a variety of care contexts. Search results were systematically screened to identify literature that addressed individual or family involvement in care planning as a primary concern, and then analysed using thematic content analysis.

**Results:**

Several themes were identified, including definitions of the concept of PDCP, essential elements of PDCP, barriers, facilitators and outcomes. The concept of PDCP is informed by multiple disciplines, including humanist philosophy, disability rights and end‐of‐life care. Essential elements of PDCP include knowing the person, integrating the person's goals in care planning and updating care plans as individuals’ needs or preferences change. Limited time for care planning in nursing homes hinders PDCP. Facilitators include regulatory mandates and humanist social trends. Outcomes of PDCP were found to be positive (e.g., increased independence), but were inconsistently assessed across studies.

**Conclusion:**

This study offers pragmatic information that can support PDCP within nursing homes and insights for policy reform that may more effectively support PDCP.

**Implications for practice:**

These findings can be used to guide implementation of PDCP.

## 1 INTRODUCTION

In nursing homes, care plans provide a guide for fundamental aspects of residents’ everyday lives, such as eating, sleeping, bathing, and dressing. In the United States, care plans are informed, in part, by the minimum data set (MDS), which is used to assess all residents on a regularly scheduled basis (Dellefield & Corazzini, 2015). Care plans identify residents’ personal and healthcare needs, the type of staff that should provide services, the frequency of services, equipment and supply needs, dietary needs and food preferences and health and personal goals. Care plans can be used by residents and their families to help structure their daily lives, by staff to plan their daily work, and by management to create operational plans (e.g., staffing protocols) that align with residents’ care plans. For long‐term residents, care plans can shape the quality of their lives for extended periods of time.

To modernise nursing home regulations, the U.S. Centers for Medicare and Medicaid Services (CMS, 2016) passed regulatory updates that require nursing homes to give residents the opportunity to direct their own care planning, as opposed to having their care plans developed for them without their input. These guidelines also specify that interdisciplinary care plan meetings should occur quarterly and include input from residents, their families, the aides who work closely with them, and food and nutrition services staff. Viewed favourably by consumer advocates, the updated care planning regulations require nursing homes to develop and implement baseline care plans within 48 hr of resident admission, learn more about the resident as a person, provide greater support for individual preferences, give residents increased control and choice, and support greater resident involvement and participation in care planning (Smetanka & Edelman, 2016). These regulations align with the culture change movement, which aims to make nursing homes more person‐centred and directed (Koren, 2010; Miller et al., 2014).

The diffusion of culture change practices is increasing, but overall nursing homes tend to implement practices that are less complex or have a more immediate cost‐benefit to the facility (Lepore et al., 2015). By contrast, implementation of PDCP transforms the traditional, provider‐driven process—of data collection, assessment, and care plan development—to a resident‐ and family‐driven process that radically shifts the fundamental care relationships among staff, residents and families. Without attention to extant knowledge of how nursing homes can effectively engage and empower residents in care planning, such facilities may fail to realise possible gains in quality of life and quality of care for residents (Sterns, Miller, & Allen, 2010).

The purpose of this study was to conduct a scoping review to develop a conceptual and pragmatic understanding of PDCP. We sought to determine how individuals or their family members are, or might be, engaged and empowered to participate in care planning in a variety of care settings. These goals are consistent with the purpose of scoping reviews, which are aimed at mapping “a wide range of literature and to envisage where gaps and innovative approaches may lie” (Ehrich, Freeman, Richards, Robinson, & Shepperd, 2002, p. 28).

### 1.1 Theoretical framework

Because PDCP is predicated on reshaping fundamental relationships in nursing homes, we drew upon the adaptive leadership framework from complexity theory to guide our scoping review, including the selection of search terms, the development of our coding scheme and the identification of themes (Corazzini et al., 2015). The adaptive leadership framework differentiates between *adaptive *and *technical *challenges, the associated adaptive versus technical work to address these challenges, and the importance of distinguishing between the two to succeed in change efforts (Corazzini & Anderson, 2014; Heifetz, Grashow, & Linsky, 2009).

Technical challenges are those for which there is a known solution. Solutions to technical challenges may be technically complex or require high‐level expertise, but addressing technical challenges is relatively straightforward within a system, with the values underlying the system remaining in place. Adaptive challenges, in contrast, are challenges with no currently‐known solution. Resultant adaptive work is more complex and may entail changing normative beliefs or values and co‐creating new solutions. Thus, addressing adaptive challenges implies openness to shifting the values that underlie a system and demanding changes to the system itself (Anderson et al., 2015; Glover, Friedman & Jones, 2002). Both technical challenges (e.g., scheduling care plan meetings at times when residents and aides can attend) and adaptive challenges (e.g., debunking the belief that nursing home residents are unable to provide useful insights regarding their care needs) are relevant to PDCP in nursing homes (Corazzini et al., 2015), and both may require collaborative work among nursing home staff, residents, and family members to develop effective solutions (Corazzini & Anderson, 2014). We drew upon this framework to understand the current conceptualisation and practices of PDCP.

## 2 METHODS

Our scoping review included peer‐reviewed research literature, editorials, reports from government regulatory agencies and consumer and industry advocacy groups. We aimed to summarise research findings related to PDCP in general (i.e., not restricted to the nursing home setting) and to identify practical examples, models, frameworks and recommendations that could inform our knowledge of how nursing homes can engage and empower residents in care planning.

### 2.1 Collecting, screening and prioritising literature

We used a systematic approach to collect literature. Firstly, a strategy for searching scientific databases was designed, including publication parameters (i.e., English language), search terms and logic (Table 1). The date range (i.e., 2010 or later) was chosen to include the body of literature published on or following the passage of H.R. 3590, the Patient Protection and Affordable Care Act, which codified person‐centred care in the U.S. healthcare system. We used this strategy to search the CINAHL, PubMed and SocIndex databases. Next, we searched for literature originating in the voluntary and advocacy sectors, and in professional and governmental organisations, using the websites of culture change organisations (e.g., Pioneer Network). We also searched the Internet using Google and identified additional websites concerned with individuals’ engagement in care planning, including the websites of government regulatory agencies and state culture change associations. Research team members also recommended literature based on their knowledge, including literature published prior to 2010. We ultimately collected 622 unique items, including peer‐reviewed and grey literature. We limited the search to papers published prior to December 2015.

**Table 1:** Scoping review search terms

Concept	Search terms
A. Person‐directed care planning	
Person‐directed	Person‐directed; Person‐centered; Person‐focused; Consumer‐driven; Consumer‐directed; Client‐driven; Client‐centered; Client‐directed; Resident‐centered; Resident‐directed; Patient‐centered; Patient‐directed; Patient activation; Patient or Resident involvement; Patient or Resident participation; Patient or Resident collaboration; Patient or Resident engagement
Care planning & assessment	Assessment; Care plan; Care goals; Care processes; Minimum Data Set; MDS; Problems; Treatment plan; Therapeutic goals; Collaborative work; Identifying challenges
B. Family engagement in care planning [Boolean logic = A and B]	
Families	Family; Informal caregiver; Care partner
Engagement	Involvement; Participation; Engagement; Collaboration; Activation
C. Socio‐contextual factors affecting resident and family engagement [Boolean logic = (C and A) or (C and B)]	
Socio‐contextual factors	Cognitive status; Dementia; Race; Ethnicity; Rural; Urban; Medicaid; Mental health or mental illness; Language

**Figure 1:**
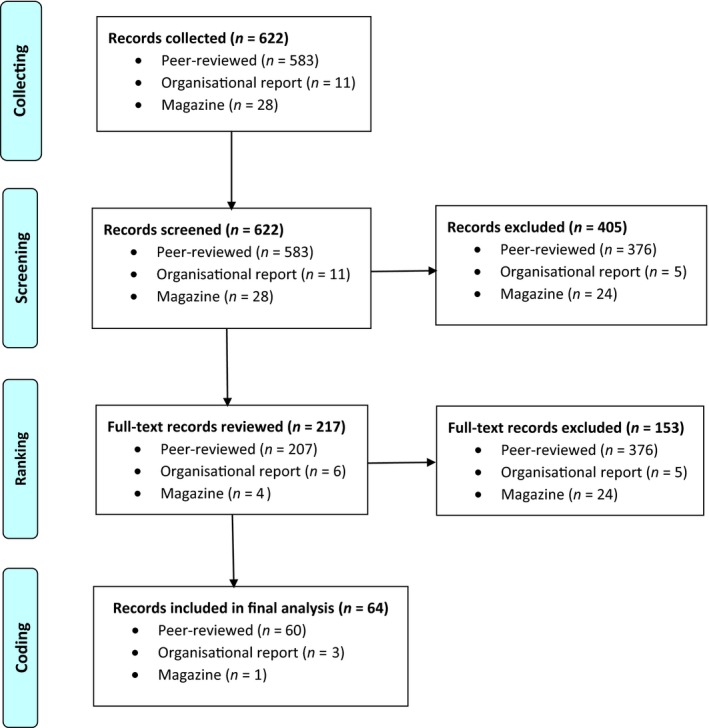
Collecting, screening and ranking literature

The inclusion criterion for further analysis of the collected literature was whether it addressed individual or family engagement in person‐directed care planning processes (in any care setting). We reviewed the 622 collected items. Some articles addressed care planning in general, but not individual or family engagement in care planning specifically; these articles were excluded from additional analysis. The research team, authors of this article, worked in pairs so that all 622 items were reviewed by at least two researchers. Members of each pair independently reviewed the abstract of each item (or the full text when no abstract was available) to determine whether the item met the inclusion criterion. The team determined that 217 items met the inclusion criterion, whereas the other 405 items did not (Figure 1).

Next, at least two researchers independently reviewed the full text of the 217 articles to identify those with primary relevance to individual or family engagement in person‐directed care planning. Items had primary relevance if individual or family engagement in planning care was a main topic; otherwise, the items were excluded from further analysis. Researchers independently reviewed each item, categorised it as having primary relevance or not, documented decisions in an Excel spreadsheet, then compared decisions. If they disagreed, they met to reach a consensus. Researchers agreed on the primary relevance of all articles. Of the 217 items screened in, 64 were categorised as having primary relevance and were then coded line‐by‐line (Figure 1).

### 2.2 Coding literature

To code the 64 primary items, we first developed a list of high‐level concepts relevant to PDCP (e.g., Implementation), key sub‐concepts (e.g., Implementation barriers), and 47 codes informed by our adaptive leadership theoretical framework. Consistent with other qualitative health services researchers (Andersen, 1995; Bradley, Curry, & Devers, 2007; Miles & Huberman, 1994), we started with a preliminary code list to build on previous insights in the field (e.g., to include codes for key adaptive leadership concepts). We uploaded the 64 items and code list to the n ‐vivo software program, which we used to record all coding. Secondly, six researchers coded the same three articles line‐by‐line, and then met to discuss and reach consensus on any code chart revisions (i.e., additions, changes or deletions). For example, the research team added a “How to” code to apply when the literature provided specific tools or strategies (i.e., “how to” guides) for achieving PDCP. The revised code list included 20 major codes and 22 subcodes. It was used to code all primary articles line‐by‐line, and no additional changes were made to the codebook (Table 2). Each primary article was coded by one researcher, and then at least one other researcher reviewed the coding. The coder and reviewer met to discuss and reach a consensus on all coding decisions. Coding was considered complete when all primary articles were coded and at least two researchers agreed on all coding decisions.

**Table 2:** Scoping review code chart

Major codes Subcodes	Description of topics to which the codes apply
Adaptive leadership	Adaptive challenges: Challenges in PDCP process which require individuals to adjust to a new situation and do the work of adapting, learning, and behavior change Adaptive leadership: Efforts to develop the capacity of individuals, family, and/or providers to address adaptive challenges in PDCP process Adaptive work: Changes in values, skills, and/or behaviors required of individuals, families, and/or providers to overcome adaptive challenges and achieve desired outcomes of PDCP process
Collaborative work	Processes whereby providers, individuals, and family members develop shared understanding of situations and solutions, both technical and adaptive; includes collaborative assessment and planning
Communication With individual With family Intraprofessional Interprofessional	Specifically addresses the issue of communication in PDCP process
Decision factors	Specifically addresses factors involved in decisions to adopt or implement PDCP
Definitions	Specifies how an author(s) defines PDCP
Engagement Individual Family	Specifically addresses how individual or family preferences are or can be incorporated in care planning processes or the care plan itself
Education	Specifies how individual, family, and/or provider is or can be prepared to engage in PDCP; may include formal and/or informal learning components
Evaluation of care plan	Specifically addresses process of and involvement in evaluation of care plan over time
Framework	A framework or conceptual model
Guidelines	Specifies PDCP in terms of or with reference to practice guidelines; may highlight consistency or challenges/contradictions
“How to”	Provides specific tools or strategies (i.e. “how to” guides) for achieving PDCP Example: When an article describes a framework and includes a list of steps to implement the elements of the framework, code as “how to”
Implementation Leadership Strategies Communication Barriers Facilitators	Specifies how PDCP is implemented This code, compared to the “how to” code, should be used with research articles that describe interventions and their implementation related to leadership, strategies, communication, barriers and facilitators. This code can also be used for non‐research articles if general implementation issues related to PDCP, outside of research, are discussed
Institutional factors Physical resources, including IT Human resources Other resources Processes	Discusses conditions for implementation of PDCP
Intervention design Individual‐level design Family‐level design Provider‐level design	Specifies design of PCDC intervention
Measures	Specific measure of PDCP. Example: When a research article or other article types describes a questionnaire or survey tool that has been used to assess individuals’ opinions or understanding of PDCP. Example: Any mention of a quality measure to assess individual/resident‐directed care
Outcomes	Specific outcomes of PDCP. This code can be used to describe a variety of achieved or related outcomes including positive or negative patient/resident, family or provider outcomes related to PDCP. One example of a positive outcome is cost savings
Policy/regulatory issues	Specifies PDCP in terms of external policy/regulatory framework; may highlight consistency or challenge/contradiction
Sociocultural factors	Specifies barriers or challenges related to PDCP for specific populations(s); population may be identified by race/ethnicity, geography, socioeconomic status, linguistic group, or other
Trajectory of care	Specifically addresses aspects of the care planning process (or factors that are relevant to the care planning process) that arise at different points along the trajectory of care (e.g., admission, 72‐hr post‐admission)

### 2.3 Identifying themes

Identification of themes began during coding. After coding 10 articles, each researcher independently reviewed all coded segments of text to identify emerging themes that conceptually summarised information in the literature. The full multidisciplinary team then met to discuss the emerging themes. The themes were reviewed, and revised or rejected, as needed. All coded segments of text were reviewed by at least two researchers to confirm themes. After all primary articles were coded, final themes were confirmed by all team members.

## 3 RESULTS

The 64 items analysed in this study included 61 peer‐reviewed journal articles, two organisational reports and one magazine article from numerous countries (Australia, Canada, Ireland, Italy, New Zealand, Norway, Scotland, Spain, Switzerland, the United Kingdom and the United States). The items included literature reviews (*n* = 18), qualitative studies (*n* = 12), quantitative studies (*n* = 11), case studies including reviews of specific models or frameworks (*n* = 11), commentaries and editorials (*n* = 7), mixed‐method studies (*n* = 3) and study protocols (*n* = 2). Publication years included one item in 2006 and 2008, seven in 2010, four in 2011, 13 in 2012 and in 2013, 19 in 2014 and six in 2015. Five overarching themes were identified from analysis of these items: (a) The Concept of PDCP, (b) Essential Elements of PDCP, (c) Barriers and Challenges to PDCP, (d) Facilitators of PDCP and (e) Outcomes of PDCP. The themes broadly characterise a variety of specific points made in the literature about individuals’ engagement in care planning (Table 3).

**Table 3:** Themes from the scoping review on person‐directed care planning (PDCP)

Theme	Description
Theme 1: The concept of PDCP	The concept of PDCP is informed by several disciplines, including humanist philosophy, disability rights, mental/behavioral health care, dementia care, end‐of‐life care and rehabilitation, including occupational, physical and pediatric rehabilitation
Theme 2: Essential elements of PDCP	Essential elements of PDCP include knowing the person, integrating the person's goals in care planning and iteratively revising care plans as the individual's needs and preferences change
Theme 3: Barriers and challenges to PDCP	Barriers and challenges to PDCP include the difficulty of changing active processes, limited time available for care planning activities, and lack of awareness among staff of the value of PDCP or how to implement PDCP
Theme 4: Facilitators of PDCP	Facilitators of PDCP include regulatory mandates and consensus statements, humanist social trends, and organizational and interpersonal factors
Theme 5: Outcomes of PDCP	Outcomes of PDCP are inconsistently assessed but commonly favorable

### 3.1 Theme 1: The concept of PDCP

Several disciplinary fields inform the concept of PDCP (Table 3). The diversity of disciplines informing the concept of PDCP was supported by our scoping review's inclusion of literature from diverse care settings (i.e., not restricted to nursing homes). These disciplines emphasise several principles that underpin a person‐directed approach to care planning: supporting autonomy, recognising personhood, and being strengths‐based.

#### 3.1.1 Supporting autonomy

Supporting autonomy—meaning that individuals are supported to make their own decisions and accept the consequences of those decisions—is foundational to PDCP. Support for individual autonomy extends back several hundred years in humanist philosophy, and it has evolved over time and varies across care contexts (Peel, 2005). Humanist social trends support patient participation in decision‐making and steer away from paternalistic models of healthcare wherein patients have commonly been passive spectators in healthcare (Longtin et al., 2010).

Contemporary considerations of autonomy in relation to older individuals in residential long‐term care (LTC) emphasise that knowing each resident and encouraging residents to make decisions about their care on a daily basis are key factors in promoting autonomy. Rodgers, Welford, Murphy, and Frauenlob (2012) emphasised the need for meaningful options in daily living. Simmons et al. (2011, p. 867) contend that nursing home residents’ autonomy supports well‐being: “[N]ursing homes that elicit and honour resident choices help to foster residents’ sense of autonomy and… their sense of well‐being. Failure to attend to choice may undermine residents’ autonomy, leading to… passivity and hopelessness.” PDCP core technical work includes eliciting and honouring resident choices.

The connection between individuals’ engagement in their care planning and their autonomy is emphasised in other care contexts. The disability rights literature describes care planning as a way of helping individuals take control of their lives and the need for care plans to be person‐centred to improve quality of life among individuals with intellectual disabilities (O'Brien & O'Brien, 2002; Smith & Carey, 2013; Smull & Sanderson, 2009). Literature on advance care planning emphasises that individuals at the end of life can autonomously make informed decisions about the care they want to receive (Hilliard, Washington, Hines, & McGill, 2013).

#### 3.1.2 Recognising personhood

The importance of planning care in a way that recognises the personhood of the individual—that is, recognises that the individual with care needs is equally human and should not be deprived of his or her personhood—is emphasised in the dementia care literature. The concepts of personhood and person‐centred care are informed by Kitwood's (1997) work on caring for individuals with dementia, and they provide a framework for nursing home staff to promote residents’ sense of identity, autonomy and agency (Brown Wilson, Swarbrick, Pilling, & Keady, 2013) The dementia care literature holds that technical components of planning care in a way that supports personhood means attending to significant events in the lives of persons with dementia and ensuring that significant details of their lives are included in care plans (Brown Wilson et al., 2013).

#### 3.1.3 Strengths‐based

Utilising an approach to care planning that attends to individuals’ strengths and capabilities rather than their weaknesses and disabilities is emphasised as foundational to planning care in a way that maximises choice for individuals receiving mental health services (Stanhope, Tondora, Davidson, Choy‐Brown, & Marcus, 2015). Taking a strengths‐based approach has promise for improving uptake and promoting effectiveness of care: “By shifting from an illness and/or deficit focus to a strengths‐based, person‐centred one… fundamentally changes a practice culture that has resulted in many people walking away from the care they need” (Stanhope et al., 2015; p. 2).

These key principles—autonomy, personhood and the strengths‐based approach—support viewing each nursing home resident as a unique individual who brings distinct and critical perspectives to care planning. They hold that each resident must be honoured as a person and incorporated as a valued partner in care planning. From the perspective of the adaptive leadership framework, such a partnership entails collaborative work between nursing home staff and residents (Corazzini & Anderson, 2014). These principles undergird the essential elements of PDCP addressed in Theme 2.

### 3.2 Theme 2: Essential elements of PDCP

The essential elements of PDCP identified by our review include knowing the individual, integrating the person's goals in care planning, and iteratively revising care plans as the individual's needs and preferences change. These essential elements of PDCP generally entail technical, rather than adaptive, changes to care planning.

#### 3.2.1 Knowing the person and integrating the person's goals in care planning

Person‐directed care planning requires that service providers get to know the person whose care plan is being developed, which is emphasised in literature on person‐centred LTC (Iris, DeBacker, Benner, Hammerman, & Ridings, 2012; Kolanowski, Van Haitsma, Penrod, Hill, & Yevchak, 2015) and on care planning among individuals with intellectual disabilities (Ames, 2013; Sanderson, 2013). For example, Ames (2013, p. 13) advises learning disability nurses to consider: “What matters to the person… What happened in the person's past that may affect who he or she is now [and]… With whom the person has important relationships.”

Specific things that service providers are encouraged to know include the individual's health status and prognosis (Gestuvo, 2012), his/her goals, preferences, needs, values, and priorities, particularly as these pertain to daily life (Haitsma, et al., 2014; Rodgers et al., 2012). Their personal histories, significant life stories, social networks (Boyd, McNabney, & Brandt, 2012), and what he or she believes will help promote recovery or optimise well‐being should also be considered (Frazee, 2011). Service providers also must know the extent to which the individual wishes to be engaged in care planning and recognise that everyone does not want to be engaged (Silow‐Carroll, Alteras, & Stepnick, 2006). The extent to which an individual may wish to be involved varies. Among veterans near the end of life, Braun, Beyth, Ford, Espadas, and McCullough (2014) identified several decision‐making styles (e.g., autonomists, altruists), each of which may require different strategies for PDCP.

Other technical work involves communicating with the individual's social network to the extent that the individual wants them to be engaged (Greene, Tuzzio, & Cherkin, 2012). For mental health services to achieve an individual's personal recovery goals, optimal care planning has been described as “a collaborative process between the service user and all of those people in the person's life whom he or she identifies as supportive of his or her recovery” (Stanhope et al., 2015, p. 2). Similarly, in the context of paediatric rehabilitation, parent‐professional collaboration is recommended (An & Palisano, 2013).

Although knowing the individual is necessary for PDCP, it is not sufficient. PDCP also requires integrating knowledge of the person's priorities and preferences into care planning (Heid et al., 2016; Iris et al., 2012). Then, the care plans can guide daily practices. Essential technical work for PDCP also includes updating care plans as the individuals’ needs or preferences change.

#### 3.2.2 Revising care plans as the individual's needs and preferences change

PDCP requires that care plans are iteratively updated as the person's needs or preferences change, and the individuals themselves should be involved in reassessing their care plans over time. To ensure that care plans remain aligned with the needs and preferences of individuals with disabilities, Sanderson (2013) suggests that practitioners must regularly ask individuals what is working and what is not working. With a focus on older people in residential care, Rodgers et al. (2012) emphasise that individuals change across the life cycle, which reinforces the importance of regularly checking with them to inform care plan updates. The iterative nature of PDCP mirrors that of adaptive leadership; according to Bailey et al. (2012, p. 7): “Adaptive leadership is fundamentally a non‐linear, iterative, reciprocal interaction between the health care practitioner and the patient.”

Although MDS assessments of nursing home residents are required on a quarterly basis, the frequency that care plans need to be updated depends, in part, on the rate at which the individual's needs or preferences change. As Haitsma, et al. (2014), p. 34) explain: “If preferences change within short periods of time, strategies are needed to assess preferences more frequently; if residents report consistent preferences, less frequent assessments may be appropriate.”

### 3.3 Theme 3: Barriers and challenges to PDCP

It takes time to get to know a person, to integrate the individual's goals in care plans, and to iteratively revise these plans as the person's needs and preferences change. Limited time for care planning and a lack of awareness of the value of PDCP are barriers across diverse care settings (Bjerkan, Vatne, & Hollingen, 2014). Furthermore, in LTC, many—perhaps most—staff are not trained in communication approaches that might engage residents in care planning (Børøsund, Ruland, Moore, & Ekstedt, 2014; Savundranayagam, 2014). The structure of nursing documentation—especially the tendency for care planning forms to require limited psychosocial detail—is likewise an obstacle (Broderick & Coffey, 2013).

The barriers of limited time and knowledge of how to engage residents in care planning are reinforced by a medical paradigm in nursing homes, whereby medical standards, resident safety and routinised care schedules take precedence over patient choice (Maurer, Dardess, Carman, Frazier, & Smeeding, 2012). Direct‐care workers are typically not involved in care planning (Kolanowski et al., 2015). This paradigm is also reflected in the broader healthcare culture where patients find it difficult to participate in discussions about their care because they are overwhelmed by too much information, and much of that in jargon they may not understand (Maurer et al., 2012). Nursing home residents in this paradigm commonly perceive facility policies as restrictive and their choices limited (Bangerter, Haitsma, Heid, & Abbott, 2015).

Similar barriers to mental health service users’ engagement in care planning have been documented. Self‐direction is a central element of the recovery model of mental healthcare wherein individuals direct their own goals, identify their preferred life paths, and determine which steps to take on that path (Onken, Craig, Ridgway, Ralph, & Cook, 2007). However, adaptive challenges to self‐direction in the care planning process have been identified, including: beliefs that recovery‐oriented practice is not unique or novel, that it is too burdensome for overextended clinicians, that it is not desired by individuals with severe mental illnesses, that it is not evidence‐based or reimbursable, that it devalues provider expertise, and that it is too risky because it increases provider exposure to incidents and liability (Stanhope et al., 2015).

Nursing homes also might find the diversity of their resident populations to be a challenge to PDCP because each resident has different preferences and capacities for engagement in care planning. Barriers to PDCP with specific groups (e.g., racial minorities) were identified in the literature, and these barriers are reinforced by the existing culture of care. For example, in some communities, it is common to view medical professionals as authoritative, which can hinder individuals from taking an active role in planning their care (Silow‐Carroll et al., 2006). Other individual‐level factors that vary across nursing home residents—their beliefs about their appropriate roles (e.g., passive vs. active) and their functional and cognitive capacities—affect their motivation, willingness, and ability to engage in PDCP (Carman et al., 2013). The issue of cognitive capacity presents particular challenges to PDCP in LTC settings where dementia—which is characterised by global cognitive deterioration, including impairments in memory, language expression and comprehension, and the ability to determine goals or carry through with plans to achieve goals—is prevalent (Moore, Boscardin, Steinman, & Schwartz, 2014).

### 3.4 Theme 4: Facilitators of PDCP

Several factors—including societal, organisational, individual and interpersonal factors—can support PDCP by helping residents and caretakers overcome technical and adaptive challenges. At the societal level, policies provide support for PDCP. For example, federal nursing home regulations updated in 2016 add residents, their families and direct caregivers to the interdisciplinary teams responsible for determining care goals (CMS, 2016). Even prior to the 2016 reform, federal guidelines made resident choice over daily schedules a right and instructed nursing home inspectors to determine whether residents were offered daily life choices (Simmons, Durkin, Rahman, Schnelle, & Beuscher, 2014). Similarly, the Patient Self‐Determination Act of 1991 advanced individuals’ abilities to participate in their own care across healthcare settings, including nursing homes (Mallers, Claver, & Lares, 2014). These policy efforts align with humanist social trends supporting patient participation in decision‐making and move away from paternalistic models of healthcare wherein patients have been passive spectators (Longtin et al., 2010). From the perspective of the adaptive leadership framework, collaborative work that empowers residents in decision‐making is essential to PDCP (Corazzini & Anderson, 2014).

Consensus statements from professional groups also support PDCP. Such consensus statements have supported the promulgation of policies that support PDCP and have offered practical guidance for PDCP implementation. For example, in the 1980 s, the Institute of Medicine (IOM, 1986) proposed nursing home regulatory reform to ensure that residents receive personalised care that attends to their physical, psychological, and social needs. This led to the Nursing Home Reform Law of 1987 and its Resident Bill of Rights, which protects the autonomy of residents to control their own money and care (Mallers et al., 2014). Advancing the transformation of healthcare culture towards patient‐directedness transnationally, the United Nations Madrid International Plan of Action on Ageing emphasises “the need to include older adults in autonomous decision‐making processes” (Welford, Murphy, Rodgers, & Frauenlob, 2012, p. 65).

At organisational and interpersonal levels, staff and residents’ families must recognise the importance of PDCP to the well‐being of residents, as such recognition can support staff to engage in PDCP (Simmons et al., 2014). From the perspective of the adaptive leadership framework, such recognition is essential to the collaborative work among nursing home residents, their families, and staff (Corazzini & Anderson, 2014).

Adoption of health information technology that supports individuals’ engagement in care planning, or communication across individuals and care providers, also facilitates PDCP (Baumann, LupPlace, & Quasey, 2010; Bjerkan et al., 2014). Using simple tools, Lankarani‐Fard et al. (2010) found that a card‐sorting game can be effective in identifying individuals’ values and priorities for end‐of‐life care.

Finally, at the individual‐level, self‐efficacy is known to help patients and families engage in their health care (Maurer et al., 2012). Notably, adaptive leadership techniques have helped individuals increase their self‐efficacy (Bailey et al., 2012; Thygeson, Morrissey, & Ulstad, 2010).

### 3.5 Theme 5: Outcomes of PDCP

Consistent with the promise for adaptive leadership to improve clinical outcomes in nursing homes (Bailey et al., 2012), several studies indicate that engaging individuals in planning their care results in improved health (Mallers et al., 2014), improved care outcomes (Haitsma, et al., 2014), greater independence in performing activities of daily living (Boltz, Resnick, Chippendale, & Galvin, 2014), and more holistic considerations of individuals’ needs (Alakeson, 2013). Asking nursing home residents about their preferences helps them feel validated, comforted and able to make choices (Haitsma, et al., 2014). Maintaining personal control contributes to health and well‐being as we age (Mallers et al., 2014). Additionally, other studies highlight the benefits of engaging individuals in communication about their care as a means of reducing the risk of miscommunication‐related adverse events (McMurray, Chaboyer, Wallis, & Fetherston, 2010).

Beneficial outcomes of PDCP are not limited to the individuals themselves. Brown Wilson et al. (2013) reported that when dementia care staff engage in conversations with residents about what is important to them, this dialogue results in improvements in the residents’ quality of care and quality of life, and promotes a sense of purpose for the caregiver. Additionally, Carman et al. (2013) reported that patient engagement can contribute to better health outcomes and improvements in quality and safety, and that if individuals then have fewer invasive treatments, healthcare costs may be reduced.

Although the many beneficial outcomes of PDCP reflect its wide‐ranging value and there is general agreement that perceived control is important for older adults’ well‐being (Simmons et al., 2014), inconsistency across studies in operationalising PDCP and examining its outcomes limits our knowledge. Furthermore, care planning interventions often are implemented in conjunction with other efforts, making it difficult to determine which changes are affecting outcomes (Stanhope et al., 2015).

## 4 DISCUSSION

Federal regulations (CMS, 2016), humanist social trends supporting patient participation in decision‐making (Longtin et al., 2010), and consensus among professional groups have called for nursing homes to support their residents in directing their own care planning (Smetanka & Edelman, 2016). The push for PDCP aligns with an established and growing body of research showing the benefits of giving individuals a say in their own care and everyday decision‐making (Haitsma, et al., 2014; Haitsma, et al., 2014; Langer & Rodin, 1976; Mallers et al., 2014; McMurray et al., 2010). Increasing support for the engagement and empowerment of nursing home residents has advanced our need for conceptual understanding of PDCP and for pragmatic information about its implementation. Because implementation remains nascent in nursing homes, this study included insights from other contexts where PDCP has been examined. This scoping review synthesises conceptual and pragmatic information about PDCP and applies information about PDCP in diverse contexts of care.

Literature addressing individuals’ engagement in their own care planning is found in several fields, including humanist philosophy, disability rights, mental/behavioural health care, dementia care, end‐of‐life care and rehabilitation. Collectively, these disciplines support the autonomy of individuals, recognise each individual's personhood, and embrace a strengths‐based approach to engaging individuals in decision‐making. These are the central goals of the nursing home culture change movement and supported by the concepts of person‐centred and person‐directed care (Lines, Lepore, & Wiener, 2015). The adaptive leadership framework brings attention to the complex challenges entailed in changing nursing home culture to enable PDCP, such as the need for both technical and adaptive changes to shift normative beliefs and values regarding the appropriate role of nursing home residents in care planning and to develop new PDCP behaviours.

For care planning in nursing homes to be person‐directed, residents must be able to determine the extent to which they engage in care planning, and facility staff must get to know the residents, integrate their goals in care planning, and iteratively revise care plans as the residents’ needs and preferences change. Technical challenges to PDCP—such as limited time available for care planning activities, the lack of awareness among staff about how to implement PDCP, and the tendency for care planning forms to require limited psychosocial detail—can hinder implementation. The adaptive leadership framework clarifies that if the right expert is engaged, such technical challenges have known solutions. These include changing nursing home reimbursement policy to cover more care planning time, increasing staff training on PDCP, and revising care plan documentation templates. However, technical solutions will not suffice for PDCP to be implemented across nursing homes. Making care planning person‐directed also will require adaptive solutions to adaptive challenges (Corazzini et al., 2015). Adaptive solutions that develop the strengths of individuals involved to facilitate changes in attitudes and skills are essential to PDCP; however, high turnover is one of multiple challenges to implementing adaptive solutions for PDCP (Donoghue, 2010). Importantly, findings indicate that PDCP promotes a sense of purpose among dementia care staff (Brown Wilson et al., 2013), which suggests it also might help improve the problem of high staff turnover, but further research is needed on how PDCP implementation impacts staff turnover, retention and other outcomes. Research on how to reform care planning practices also could contribute valuable information for supporting nursing homes to make care planning person‐directed.

Because this review included literature from varied care contexts, the findings are not all specific to nursing home populations. The essential elements of PDCP identified in this study (e.g., supporting autonomy) could be applied to nursing home residents, but the specific approaches would need to be tailored to each individual's needs and preferences. Likewise, these findings suggest that specific organisational and policy changes may be needed to better implement PDCP in nursing home settings—such as changes to care planning tools, processes, and reimbursement. These potential changes require further attention. Furthermore, the themes identified in this scoping review were informed by literature that was mostly published between 2010 and 2015, but a more comprehensive literature review on PDCP including earlier and more recent publications would contribute additional information that could be used to further validate and expand upon the themes we identified. The review also was limited by a focus on resident involvement in care planning and did not explicitly attend to resident involvement in their assessments (e.g., MDS assessments), which provide foundational information for their care plans. A more complete understanding of PDCP requires research on resident involvement in their assessments.

For nursing home leaders seeking to implement PDCP, findings from this study indicate that it is essential to establish processes to engage residents in care planning and to iteratively update care plans per residents’ preferences. Allotting ample time for resident engagement in care planning can be a challenge. Findings suggest that policies—such as requirements for care plan meetings, their frequency and the amount of time that providers are reimbursed for care planning—can have an impact on implementation of PDCP.

## 5 CONCLUSION

This study helps to clarify the meaning of PDCP by highlighting the diverse disciplinary foundations of this concept and identifying its essential elements. It advances understanding of PDCP implementation, including barriers, facilitators, and outcomes. PDCP addresses federal requirements that nursing home residents be the locus of control in their care planning and coincides with trends in the nursing home industry towards person‐centred practices. Despite broad‐based momentum to empower nursing home residents to direct their own care planning, there are significant and complex challenges, both technical and adaptive, that can deter implementation. Addressing the limited time allotted for care planning in nursing homes is a financial decision (e.g., reimbursement for care planning), as well as an operational one (e.g., nursing home staffing and work plans). Aligning reimbursement policies and nursing home operations with values that honour the autonomy of nursing home residents in care planning may be critical for these individuals to be broadly engaged and empowered.

**IMPLICATION FOR PRACTICE**

- This study helps to clarify the meaning of person‐directed care planning by highlighting its diverse disciplinary foundations and identifying its essential elements for implementation in nursing homes.
- This study advances the understanding of person‐directed care planning by identifying barriers and challenges to implementation, as well as facilitators and outcomes of person‐directed care planning.

- To implement person‐directed care planning in nursing homes, facility staff must get to know older individuals, integrate their goals in care planning, and revise care plans as their goals change.
- Implementation of person‐directed care planning can support numerous improvements in nursing home outcomes, including improved health, improved care outcomes, and improvements in older individuals’ performance of daily activities.

- Findings indicate that changing nursing home reimbursement policies to cover more care planning time may be needed for person‐directed care planning to be widely implemented in nursing homes.
- For widespread implementation of person‐directed care planning, findings indicate that nursing home staff need training on person‐directed care planning and, specifically, communication approaches for engaging residents in care planning.
